# Pediatric lymphomatoid papulosis: a broad clinicopathologic spectrum including rare variants in a single-center case series

**DOI:** 10.3389/fmed.2026.1948975

**Published:** 2026-09-29

**Authors:** Margherita Pelucchi, Andrea Michelerio, Elif Tamay Gul, Alessandro Svizzero, Elisa Curto, Giuseppe Neri, Marco Paulli, Valeria Brazzelli

**Affiliations:** 1 Dermatology Unit, Fondazione IRCCS Policlinico San Matteo, Pavia, Italy; 2 Department of Clinical-Surgical, Diagnostic and Pediatric Sciences, University of Pavia, Pavia, Italy; 3 Pathology Unit, Fondazione IRCCS Policlinico San Matteo, Pavia, Italy; 4 Department of Molecular Medicine, University of Pavia, Pavia, Italy

**Keywords:** lymphomatoid papulosis, pediatric patients; cutaneous CD30-positive lymphoproliferative disorders, histopathology; immunohistochemistry, T-cell lymphoma

## Abstract

**Background:**

Lymphomatoid papulosis (LyP) is a rare CD30-positive cutaneous T-cell lymphoproliferative disorder, extremely uncommon in children and adolescents. Despite its indolent course, LyP may mimic malignant lymphomas clinically and histopathologically, making diagnosis challenging in pediatric patients.

**Objectives:**

To characterize the clinical, histopathologic, immunophenotypic and molecular features of pediatric LyP and to describe treatment approaches and clinical outcomes.

**Methods:**

We performed a retrospective observational study of pediatric patients with clinicopathologically confirmed LyP evaluated at our tertiary referral center between 2004 and 2025. Demographic, clinical, histopathologic, immunophenotypic, molecular, treatment and follow-up data were collected.

**Results:**

Nine patients were included (8 males, 1 female), with a median age at diagnosis of 11 years (range 2–18). Lesions predominantly involved the limbs (9/9, 100%), followed by the trunk (5/9, 56%) and hands (4/9, 44%). Ulceration or necrotic evolution occurred in 6/9 patients (67%). Type A was the most common histologic subtype (5/9, 56%), followed by single cases of types B, D, E and one overlap A–C case. CD30 expression was observed in all patients. T-cell receptor clonality was identified in 4/5 tested cases. Management was mainly conservative, consisting of topical corticosteroids and phototherapy (nbUVB or PUVA). Methotrexate was used in one patient. At the last follow-up, all patients were free of active LyP lesions, and no associated hematologic malignancy was documented.

**Conclusions:**

Pediatric LyP shows heterogeneous clinicopathologic features with overall favorable outcomes. However, long-term follow-up remains warranted due to the lifelong risk of associated lymphoproliferative disorders.

## Introduction

Lymphomatoid papulosis (LyP) is a rare primary cutaneous CD30-positive T-cell lymphoproliferative disorder included in the current World Health Organization (WHO) classification of lymphoid neoplasms (1, 2). Its incidence is estimated at 1.2–1.9 cases per million person-years and pediatric-onset disease is extremely uncommon (2, 3).

LyP presents with recurrent crops of papules or nodules that spontaneously heal over several weeks, often leaving varioliform, atrophic, or hypopigmented scars. Although clinically indolent, LyP carries a lifelong risk of associated lymphoid neoplasms and may display histopathologic features that overlap with cutaneous lymphomas, making diagnosis particularly challenging in pediatric patients (4–6). It is characterized by a polymorphous infiltrate containing atypical lymphoid cells with variable CD30 expression within an inflammatory background (7). The current WHO-EORTC classification recognizes five classical clinicopathologic subtypes (A–E), together with the recently described DUSP22/IRF4-rearranged variant (1, 7–9).

Despite increasing recognition of pediatric LyP, available evidence remains limited. We present a retrospective single-center study of nine pediatric LyP patients, focusing on their clinical presentation, histopathologic and molecular findings, therapeutic approaches, and outcomes.

## Methods

We conducted a retrospective observational study of all consecutive patients aged ≤18 years at diagnosis with clinicopathologically confirmed LyP evaluated at our tertiary referral center between 2004 and 2025. Inclusion criteria required available diagnostic biopsy material, ≥1 year of follow-up, and adequate clinical and follow-up data. Of 13 potentially eligible patients, four were excluded due to incomplete data, leaving nine for analysis.

Clinical data were retrieved from medical records and included demographics, lesion morphology and distribution, laboratory findings, virologic studies (serology and/or molecular testing), systemic staging, treatment, and clinical outcomes. All histopathologic specimens were re-reviewed by two dermatopathologists with dedicated expertise in cutaneous lymphomas (M.P. and G.N.), who assigned the histopathologic subtype by consensus, according to the current WHO–EORTC classification; DUSP22/IRF4 rearrangement analysis was not performed in this cohort. TCR clonality was assessed on skin-biopsy specimens using PCR/capillary electrophoresis or NGS, according to the methodology available at the time of testing, targeting the TCR*γ* and/or TCR*β* loci. Disease course was categorized descriptively as self-limited (a single documented episode during follow-up), relapsing-remitting (multiple recurrent episodes with documented lesion-free intervals) or chronic-relapsing (persistent or frequently recurrent disease without documented lesion-free intervals). Follow-up duration was calculated from diagnosis to the last available visit.

Clinical status at the most recent follow-up was defined by the absence or presence of active LyP lesions, irrespective of residual post-inflammatory skin changes. Assessment was clinical; histopathologic confirmation was not performed, as resolved lesions were not re-biopsied. Data were collected within the institutional cutaneous T-cell lymphoma registry (CUTLY protocol), approved by the local Institutional Review Board (N./D.G./1302, 01/10/2023, Protocol 0051133/23), which includes retrospective and prospective data collection. Informed consent for the research use of clinical data was obtained from patients and/or their parents or legal guardians, as applicable, according to institutional procedures, with dedicated written consent at enrollment for prospective cases. The study was conducted in accordance with the Declaration of Helsinki.

## Results

Nine patients with LyP were identified between 2004 and 2025 (Tables 1, 2). The cohort included eight males and one female. Age at diagnosis ranged from 2 to 18 years (mean 9.8 years; median 11 years).

**Table 1:** Clinical characteristics, lesion distribution, virologic findings, treatment and clinical course of the nine pediatric patients with LyP included in the study.

Case	Sex/age at diagnosis, y	Follow-up, y	Lesion distribution	Clinical presentation	Virologic findings	Treatment	Clinical course
1	M/7	3	Buttocks, trunk, limbs	Multiple papules with residual scarring	ND	nbUVB	Relapsing-remitting
2	M/14	1	Trunk, neck, hands, limbs	Multiple papulonodular lesions	ND	Topical steroids; nbUVB	Self-limited
3	M/11	5	Diffuse, predominantly involving the limbs	Papulonodular lesions with crusting and ulceration	HHV-6 DNA positive (1,710 copies/mL)	Topical steroids; nbUVB; MTX (10 mg/week)	Relapsing-remitting, with one recurrence after MTX dose reduction
4	M/2	3	Trunk, limbs	Erythematous nodules, some with ulceronecrotic center	CMV IgG positive; EBV EBNA IgG positive; Parvovirus B19 IgG positive; HHV-6 DNA positive (25,200 copies/mL)	Topical steroids	Chronic-relapsing
5	F/3	4	Limbs, buttocks	Firm nodules with central ulceration and surrounding erythema	EBV EBNA IgG positive; HHV-6 DNA positive (1,035 copies/mL); HHV-7 DNA positive (186,120 copies/mL)	Topical steroids	Relapsing-remitting
6	M/11	3	Limbs, hands	Multiple erythematous papules and nodules, with ulcers and crusts	CMV IgG positive; HHV-7 DNA positive (1,530 copies/mL)	Topical steroids; topical retinoids; nbUVB	Relapsing-remitting
7	M/18	>20	Trunk, limbs, hands, face	Erythematous-violaceous papulonodular lesions	ND	PUVA/nbUVB	Initially self-limited, followed by a single severe recurrence
8	M/12	1	Limbs, hands, face	Erythematous papules with necrotic evolution at different stages	HHV-7 DNA positive (27,090 copies/mL)	Topical steroids; nbUVB	Self-limited
9	M/10	1	Diffuse, predominantly involving the trunk and limbs	Erythematous papulopurpuric lesions with crusting and ulceration, with residual scarring and hypopigmented patches.	CMV IgG positive; EBV EBNA IgG positive; Parvovirus B19 IgG positive; HHV-7 DNA positive (6,390 copies/mL)	Topical steroids; nbUVB	Chronic-relapsing

No active LyP lesions were documented at the last available visit in any patient.

ND, not done; nbUVB, narrowband ultraviolet B; PUVA, psoralen plus ultraviolet A; MTX, methotrexate; CMV, cytomegalovirus; EBV, Epstein–Barr virus; EBNA, Epstein–Barr nuclear antigen; HHV, human herpesvirus.

**Table 2:** Histopathologic, immunophenotypic, and molecular features of the nine pediatric patients with LyP included in the study.

Case	Histopathologic features	Predominant immunophenotype	LyP subtype	TCR clonality/ancillary studies	No. of biopsies
1	Superficial polymorphous infiltrate extending from the dermis to the hypodermis, composed of small, atypical lymphocytes with focal epidermotropism and rare blast-like CD30 + cells	CD3+, CD5+, CD8-; rare CD30 + cells	B	Not performed	2
2	Patchy polymorphous dermal infiltrate with scattered large CD30 + blast-like cells in a mixed inflammatory background; mild epidermal acanthosis and spongiosis	CD30+; CD2+, CD3+, CD4+, CD5+, partial CD7 loss; CD8-, TIA-1-, ALK-	A	Not performed	1
3	Psoriasiform epidermal hyperplasia with spongiosis and dense dermal infiltrate including eosinophils and neutrophils, with isolated perifollicular blast-like cells	CD30+/-, MUM1+/-; CD2+, CD3+, CD4+/-, CD5+/-, CD7+/-, CD8+/-; Ki-67 ∼10%	A	Monoclonal TCR*γ* rearrangement detected	2
4	Dense angiocentric and angioinvasive infiltrate composed of small, atypical lymphocytes, large CD30 + cells, numerous histiocyte-macrophages, and scattered Langerhans cells	CD30+ ∼20%; CD8+; granzyme B+, perforin+; CD3+, CD5+, CD7+; BF1+; ALK-	E	Monoclonal TCR*β* and TCRγ rearrangements detected; EBER *in situ* hybridization negative	1
5	Psoriasiform epidermal hyperplasia with central ulceration and wedge-shaped polymorphous dermal infiltrate containing atypical CD30 + cells, neutrophils, and eosinophils	CD30+; CD2+, CD3+, CD4+, CD5+; perforin+/-; ALK-	A	Not performed	2
6	Wedge-shaped polymorphous infiltrate with large atypical CD30 + cells and focal sheet-like growth; marked epidermotropism	CD30+; CD2+/-, CD3+/-, CD4+, CD5+/-, CD7-; CD8-; granzyme B+, perforin+; Ki-67 moderately increased	A/C	Clonal TCRγ rearrangement involving Vγ1, Vγ2, and Jγ regions	2
7	Perivascular and peri-adnexal polymorphous infiltrate with scattered large CD30 + cells in a mixed inflammatory background, including histiocytes and B cells; pseudoepitheliomatous hyperplasia	CD30+ ∼10%; CD3+, ALK-; TIA-1 +/-; LAT+	A	Not performed	2
8	Epidermotropic polymorphous lymphocytic infiltrate with basal aggression and pagetoid spread; focal wedge-shaped distribution	CD30+; CD2+/-, CD3+, CD5+/-; CD8+; granzyme B+, perforin+; ALK-	D	No clonal TCR rearrangement detected	1
9	Wedge-shaped T-cell infiltrate with perivascular and peri-adnexal involvement; irregular epidermal acanthosis and spongiosis	CD30+, MUM1+; CD2+/-, CD3+, CD4+/-, CD5+, CD7+/-, CD8+/-; granzyme B+	A	Dominant monoclonal pattern on a polyclonal background	1

ALK, anaplastic lymphoma kinase; BF1, Beta Framework 1; EBER, Epstein–Barr virus-encoded RNA; LAT, linker for activation of T cells; LyP, lymphomatoid papulosis; MUM1, multiple myeloma oncogene 1; TCR, T-cell receptor; TIA-1, T-Cell Intracellular Antigen 1.

Cutaneous lesions involved the limbs in all patients (Figure 1A). Additional sites included the trunk (5/9, 56%), hands (4/9, 44%), gluteal region (2/9, 22%), and face (2/9, 22%). Two patients showed diffuse involvement, while no mucosal lesions were documented. Lesions ranged from 2 to 3 mm papules to papulonodular lesions up to 1 cm in diameter. Ulceration, crusting, or necrotic evolution occurred in 6/9 patients (67%), including both children younger than 5 years. Residual scarring and/or pigmentary changes were observed in two patients (Cases 1 and 9), first documented 7 and 9 months after diagnosis, respectively, and persisting at the last follow-up.

**Figure 1:**
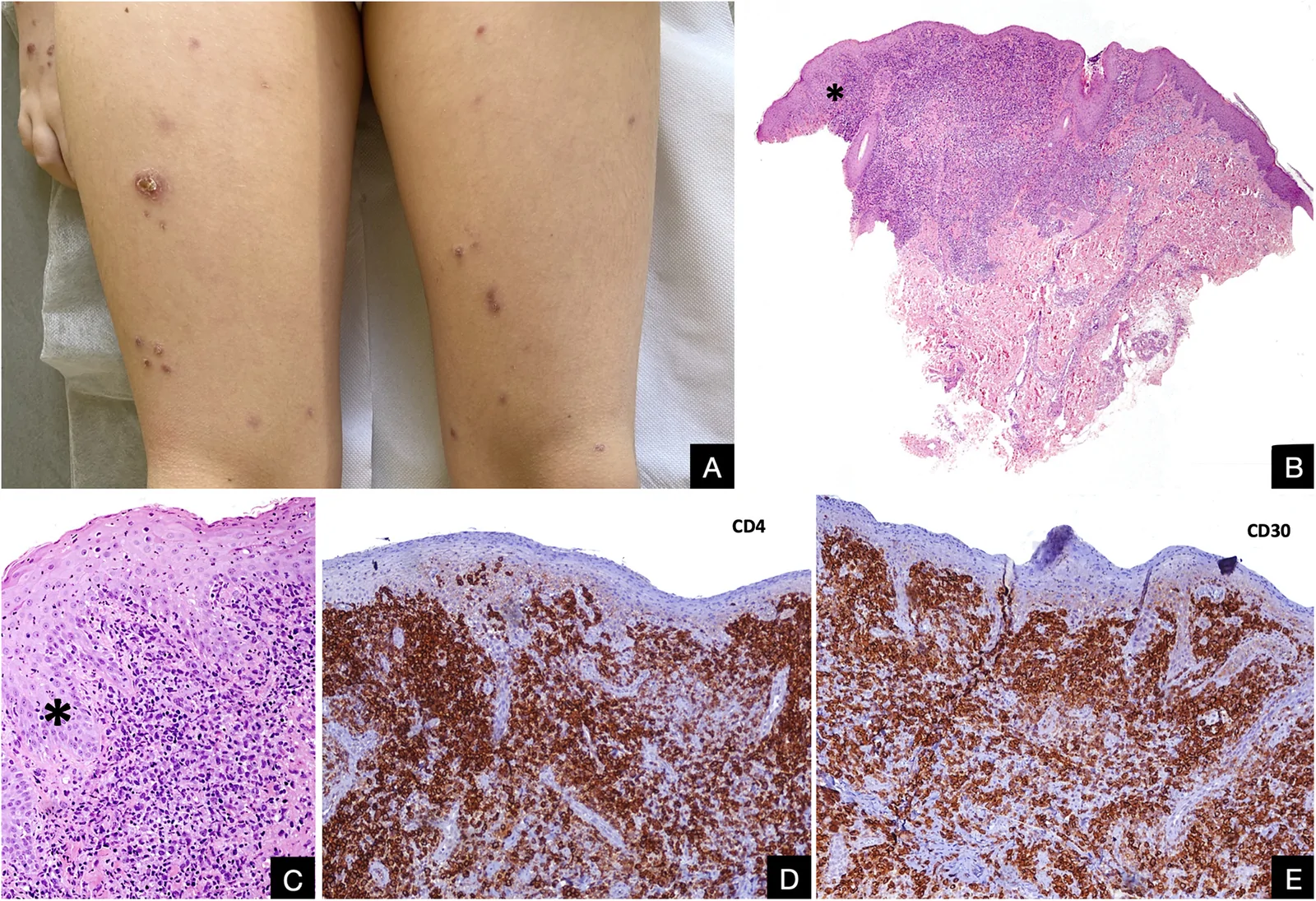
Case 6. Eleven-year-old boy with lymphomatoid papulosis showing overlapping type A and type C features. **(A)** Multiple erythematous papules and nodules with focal ulceration and crusting on the lower limbs. **(B)** Wedge-shaped polymorphous dermal infiltrate (H&E, ×20). **(C)** Medium-to-large atypical lymphoid cells with focal epidermotropism and focal sheet-like growth (H&E, ×100). **(D)** Predominance of CD4-positive T cells with epidermal infiltration (CD4 immunohistochemical stain, ×100). **(E)** CD30 expression in atypical lymphoid cells (CD30 immunohistochemical stain, ×100).

Baseline complete blood count and routine biochemical tests were unremarkable in all patients. Serum LDH levels were within the reference range in 8/9 patients; with a mild elevation in one (Case 1, 496 U/L; upper reference limit 460 U/L). Virologic investigations were available in six patients, showing IgG positivity for CMV (3/6), EBV EBNA (3/6), and parvovirus B19 (2/6). Blood PCR detected HHV-6 DNA in 3/6 and HHV-7 DNA in 4/6, including one with concurrent positivity. In one patient, Epstein–Barr virus *in situ* hybridization (EBER) on a skin biopsy specimen was negative.

Histopathologic findings were heterogeneous (Table 2). Type A was the most frequent subtype (5/9, 56%), followed by single cases of type B, type D, and type E (Figure 2); one patient showed overlapping type A/C features (Figures 1B–E). CD30 expression was detected in all cases, although one showed only partial and variable expression. T-cell receptor (TCR) gene rearrangement analysis was available in five patients and showed monoclonal TCR*γ* rearrangement or a dominant monoclonal pattern in 4/5 (80%), including one case with combined TCR*β* and TCR*γ* clonality. Serial biopsies were obtained in 5/9 patients (56%).

**Figure 2:**
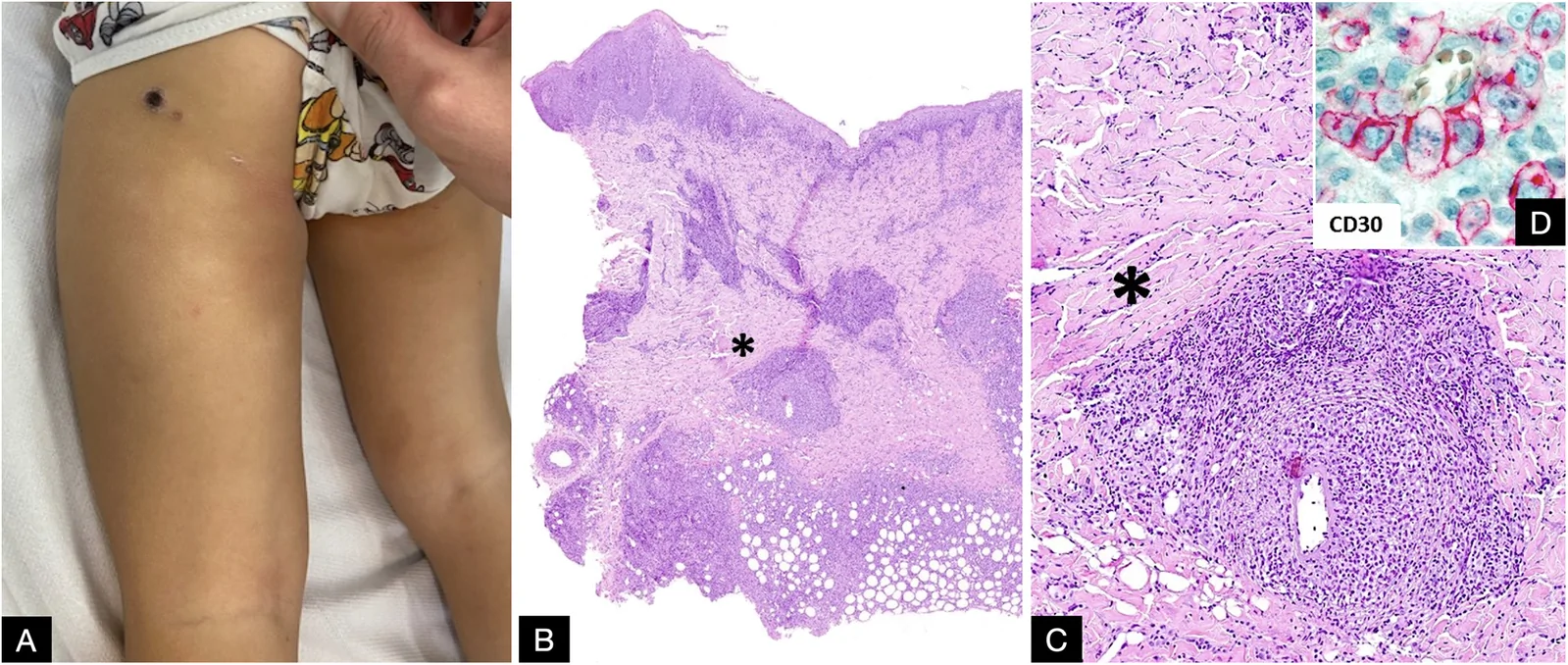
Case 4. Two-year-old boy with type E lymphomatoid papulosis. **(A)** Clinical image showing a necrotic papulonodular lesion on the thigh, with a smaller adjacent erythematous papule. **(B)** Deep dermal lymphoid infiltrate with prominent angiocentric distribution (H&E, ×20). **(C)** Angiocentric pleomorphic lymphoid infiltrate surrounding and involving a dermal vessel (H&E, ×100). **(D)** CD30 expression in atypical lymphoid cells (CD30 immunohistochemical stain, ×600).

Staging investigations were performed selectively according to clinical indication: peripheral lymph node ultrasound was performed in 2/9 patients (Cases 3 and 9) for the assessment of lymphadenopathy, whereas contrast-enhanced CT together with 18F-FDG PET was performed in 1/9 patient (Case 1) based on the initial clinical assessment. No extracutaneous disease was identified.

Management was predominantly conservative: nbUVB was administered in 7/9 patients, topical corticosteroids in 7/9, and topical retinoids in 1/9. Low-dose methotrexate (10 mg/week) was used in 1/9 patient, whereas another patient received combined PUVA/nbUVB phototherapy.

Follow-up ranged from 1 year to more than 20 years (median 3 years, IQR 1–4 years). Two patients showed a self-limited course, four a relapsing-remitting pattern, two a chronic-relapsing course, and one an initially self-limited course followed by a single severe recurrence. At the last visit, all patients were free of active LyP lesions. No associated hematologic malignancy was documented during follow-up.

## Discussion

Lymphomatoid papulosis (LyP) is a rare CD30-positive lymphoproliferative disorder, with pediatric cases accounting for only 2%–9% of all diagnoses (2, 3). The demographic characteristics of our cohort were consistent with previous pediatric series (2, 3, 10). The mean age at diagnosis (9.8 years) closely mirrored published data, whereas the marked male predominance observed in our cohort (M:F 8:1) exceeded that reported in larger studies and likely reflects the limited sample size (2, 3, 10).

The clinical presentation of our patients fell within the recognized spectrum of pediatric LyP. Lesions predominantly involved the extremities, with additional involvement of the trunk, face and gluteal region. This distribution is similar to that reported in adults, suggesting no distinctive site predilection in childhood (4, 7). No mucosal lesions were observed, although mucosal LyP is a rare but recognized presentation, including in pediatric type E disease (7, 11).

A notable finding was the high frequency of ulcerative, crusted or necrotic lesions, observed in 6/9 patients (67%), including both children younger than 5 years. Ulceration is a recognized manifestation of LyP and is particularly characteristic of type E, although it may also occur in cytotoxic type D lesions (2, 12, 13). Pediatric cohorts rarely provide age-stratified data on ulceronecrotic presentations, making comparisons difficult (2). In this context, the apparent clustering of this manifestation in very young children in our series should be considered hypothesis-generating and warrants further investigation.

Diagnostic uncertainty remains a major challenge in pediatric LyP. In our series, serial biopsies were obtained in 5/9 patients (56%). In these cases, the initial specimens, frequently sampled from early papular lesions, showed non-specific or incomplete features that did not fulfil the diagnostic criteria despite a clinically suggestive presentation, so that a further biopsy from a more developed lesion was needed to confirm the diagnosis. Age at first lesion onset and the interval from onset to diagnosis were not systematically recorded, and our data are therefore not directly comparable with the mean diagnostic delay of 1.3 years and the initial misdiagnosis rate of 26% reported in the largest contemporary multicenter pediatric cohort (2).

The current WHO classification recognizes five classical histologic subtypes of LyP (A–E), together with a more recently described DUSP22/IRF4-rearranged variant (1, 7, 8). Our cohort covered a broad histopathologic spectrum, with type A predominating, alongside single cases of types B, D, and E, and one overlap A/C case. This distribution is broadly consistent with the literature, in which type A accounts for approximately 70%–75% of cases in large adult series and 55% in the EORTC pediatric cohort (2, 7).

Particularly noteworthy was the presence in our cohort of both type D and E LyP. Although uncommon, these variants are clinically relevant because they may present with ulceration, necrosis, purpura, or rapidly progressive lesions and can closely mimic aggressive cytotoxic lymphomas (10, 12–18). In large adult-based registries, type D and E account for approximately 4%–5% and 3%–4% of cases, respectively (14–16). Pediatric data remain limited and differ appreciably: the EORTC pediatric cohort reported a substantially higher proportion of type D (17%) and no cases of type E (2, 10). Their apparent rarity should be interpreted cautiously, as both variants were recognized as distinct clinicopathologic entities only relatively recently and may therefore have been underrecognized in older series. Moreover, available data on these subtypes remain largely derived from adult-oriented cohorts, while pediatric cases are only sporadically reported and predominantly involve adolescents and older children (10, 17, 18). Accordingly, our patient, diagnosed at 2 years of age, may represent one of the youngest reported cases of type E LyP.

Clinicopathologic correlation was essential to distinguish LyP from its main histologic mimickers, particularly in cases showing overlapping or cytotoxic features. In the A/C overlap case, the recurrent, spontaneously regressing papulonodular lesions favored LyP despite focal sheet-like CD30 + growth, while in the D and E variants the self-healing course, absence of systemic or mucosal involvement, and negative staging supported LyP over aggressive cytotoxic lymphomas (12, 13, 15).

CD30 expression was detected in all cases, although partial staining was observed in one patient classified as LyP type A. Reduced or absent CD30 expression is more commonly reported in type B lesions; however, occasional discordant findings have been described in otherwise typical LyP, particularly among different lesions or at different disease stages. In our case, the diagnosis was supported by the characteristic clinical course and histopathologic findings (7, 19).

Molecular analysis further supported the lymphoproliferative nature of the lesions. T-cell receptor (TCR) rearrangement studies demonstrated monoclonality or a dominant clonal pattern in 4/5 tested patients (80%), consistent with the 77% reported rate (2). The absence of detectable clonality does not exclude the diagnosis and may reflect lesion stage, sampling variability, inflammatory background, or technical factors, including differences in sensitivity between PCR- and sequencing-based assays, which have been documented in other cutaneous T-cell lymphomas (20).

Virologic investigations, although performed non-systematically, documented prior viral infection and/or detectable viral DNA, with a notable representation of HHV-6 and HHV-7. Although the etiopathogenesis of LyP remains incompletely understood, the lymphotropic nature and latent behavior of roseoloviruses provide a biologically plausible rationale for investigating their possible association with CD30-positive cutaneous lymphoproliferative disorders (7, 21). Nevertheless, these findings should be regarded as exploratory and do not establish an etiologic or pathogenic role for HHV-6/7 in LyP. Interpretation of HHV-6/7 PCR positivity requires caution, particularly in children, in whom latent infection and asymptomatic viral persistence are common (22–24).

Systemic staging was selectively performed and consistently negative, supporting the rarity of extracutaneous involvement in pediatric LyP and current international recommendations advocating a clinically guided approach rather than routine extensive work-up (25). Additional evaluation should be reserved for persistent lymphadenopathy, B symptoms, or atypical clinical features.

Management was largely conservative, relying primarily on topical corticosteroids and phototherapy, with systemic treatment reserved for severe or refractory disease. This approach is consistent with current recommendations, which focus on symptom control and quality of life, as no available therapy has been shown to modify the natural history of LyP or eliminate the risk of associated lymphoid neoplasms (25). nbUVB was used in most of our patients aged ≥5 years because of its favorable safety profile, while PUVA was reserved for a single adolescent with longstanding disease. When systemic therapy is required, low-dose MTX is commonly used for disease control, although relapse after treatment discontinuation is common, with reported rates of at least 40% (25).

The retrospective single-center design, small sample size, non-standardized treatments, and heterogeneous, often limited follow-up represent the main limitations of this study and preclude firm conclusions regarding comparative treatment efficacy or long-term malignancy risk. Nevertheless, our findings underscore the marked clinical and histopathologic heterogeneity of pediatric LyP, including ulceronecrotic presentations, uncommon histologic variants, and diagnostic complexity. Awareness of this broad spectrum and careful clinicopathologic correlation are essential to avoid diagnostic delay and unnecessary interventions. Despite its generally indolent course, long-term surveillance remains warranted because of the lifelong risk of associated lymphoid neoplasms (4–6).

## Data Availability

The raw data supporting the conclusions of this article will be made available by the authors, without undue reservation.
